# Psycho-socioeconomic bio-behavioral associations on all-cause mortality: cohort study

**DOI:** 10.15171/hpp.2016.12

**Published:** 2016-06-11

**Authors:** Paul D. Loprinzi, Robert E. Davis

**Affiliations:** ^1^Jackson Heart Study Vanguard Center of Oxford, Center for Health Behavior Research, Physical Activity Epidemiology Laboratory, Department of Health, Exercise Science and Recreation Management, The University of Mississippi, University, MS 38677, USA; ^2^Center for Health Behavior Research, Physical Activity Epidemiology Laboratory, Department of Health, Exercise Science and Recreation Management, The University of Mississippi, University, MS 38677, USA

**Keywords:** Behavior, Biological, Epidemiology, Health, Psychology, Survival

## Abstract

**Background: ** The purpose of this study was to examine the cumulative effects of psychological,socioeconomic, biological and behavioral parameters on mortality.

**Methods:** A prospective design was employed. Data from the 2005-2006 National Health and Nutrition Examination Survey (NHANES) were used (analyzed in 2015); follow-up mortality status evaluated in 2011. Psychological function was assessed from the Patient Health Questionnaire-9 (PHQ-9) as a measure of depression. Socioeconomic risk was assessed from poverty level, education, minority status, and social living status. Biological parameters included cholesterol, weight status, diabetes, hypertension and systemic inflammation. Behavioral parameters assessed included physical activity (accelerometry), dietary behavior, smoking status (cotinine) and sleep. These 14 psycho-socioeconomic bio-behavioral (PSBB) parameters allowed for the calculation of an overall PSBB Index, ranging from 0-14.

**Results: ** Among the evaluated 2530 participants, 161 died over the unweighted median follow-up period of 70.0 months. After adjustment, for every 1 increase in the overall PSBB index score,participants had a 15% reduced risk of all-cause mortality (HR = 0.85; 95% CI: 0.76-0.96). After adjustment, the Behavioral Index (HR = 0.73; 95% CI: 0.60-0.88) and the Socioeconomic Index(HR = 0.82; 95% CI: 0.68-0.99) were significant, but the Psychological Index (HR = 0.67; 95%CI: 0.29-1.51) and the Biological Index (HR = 1.03; 95% CI: 0.89-1.18) were not.

**Conclusion:** Those with a worse PSBB score had an increased risk of all-cause mortality.Promotion of concurrent health behaviors may help to promote overall well-being and prolong survival.

## Introduction

Arguably, various *psychological* (e.g., depression), *socioeconomic* (e.g., poverty), *biological* (e.g., diabetes) and *behavioral* (e.g., physical activity) parameters play an important role in promoting and regulating health and preventing premature mortality. Indeed, psychological (e.g., depression), socioeconomic (e.g., poverty), biological (e.g., cardiovascular disease risk factors, such as cholesterol) and behavioral (e.g., physical activity and diet) parameters have all been shown to independently associate with mortality.^1-8^ Less common in the literature, however, is the cumulative effects of these parameters on all-cause mortality. Here, in this study we comprehensively examine the potential cumulative and independent effects of these parameters on all-cause mortality, hereafter referred to as the psycho-socioeconomic bio-behavioral (PSBB) index. That is, we evaluate the independent contributions of psychological, socioeconomic, biological and behavioral parameters on mortality risk. With regard to the cumulative associations, we evaluate whether individuals who have multiple unfavorable levels within each of these 4 categories have the highest mortality risk. Identification of individual and cumulative effects of these parameters on mortality risk may help practitioners effectively promote lifestyle strategies to improve health and reduce early mortality risk among adults.

## Materials and Methods

### Study design and participants

The present study was a prospective cohort study. Data were used from the 2005-2006 National Health and Nutrition Examination Survey (NHANES).^9^ This NHANES cycle was evaluated as this is the only NHANES cycle that included all PSBB parameters assessed herein. In the sample, 2530 participants provided data on the study variables.

The NHANES is an ongoing survey conducted by the Centers for Disease Control and Prevention (CDC) that uses a representative sample of non-institutionalized US civilians selected by a complex, multistage, stratified, clustered probability design. For the mortality analyses, data from participants in the 2005-2006 NHANES cycles were linked to death certificate data from the National Death Index. Person-months of follow-up were calculated from the date of the interview until date of death or censoring on December 31, 2011, whichever came first.

### Measurement of psycho-socioeconomic bio-behavioral influences

Psychological

The only available psychological parameter among adults of all ages in the 2005-2006 NHANES was an assessment of depression. Participants completed the Patient Health Questionnaire-9 (PHQ-9) during the computer-assisted personal interview.^10^ The PHQ-9 is the 9-item depression module from the full PHQ. The PHQ-9 depression scale consists of the actual 9 criteria upon which the diagnosis of DSM-IV depressive disorders is based. Sample items include, ‘over the last two weeks, how often have you been bothered by’: “feeling down, depressed or hopeless,” “feeling tired or having little energy,” and “trouble concentrating on things, such as reading the newspaper or watching television.” For each question, participants responded using a 4-point Likert scale, with responses including *not at all* (0), *several days* (1), *more than half the days* (2), and *nearly every day* (3). Items were summed, with higher scores indicating greater severity of depression. As a measure of severity, the PHQ-9 can range from 0 to 27, since each of the 9 items can be scored from 0 (not at all) to 3 (nearly every day). The PHQ-9 has demonstrated evidence of reliability and validity, with Cronbach alpha ranging from 0.86-0.89 and a 48-hour test-retest correlation coefficient of 0.84.^10^ In the present sample, internal consistency of this questionnaire, as measured by Cronbach alpha, was 0.81. Consistent with other studies, participants were considered to have moderate or greater depression if they had a PHQ-9 score of 10 or higher.^10^

*Socioeconomic*

Consistent with other studies,^11^ socioeconomic risk was assessed from 4 NHANES variables, namely poverty level, education, minority status, and social living status. For poverty level, participants were dichotomized into being above or below the poverty level, with an income-to-poverty ratio of <1 denoting below the poverty threshold. For education, participants were dichotomized as <12th grade education or 12th grade or higher education. For minority status, participants were classified as either non-Hispanic white or other (Mexican American, other Hispanic, non-Hispanic black, or other race). Lastly, for social living status, participants were classified as married/living with a partner or other (widowed, divorced, separated or never married).

Biological

Biological parameters included cholesterol, weight status, diabetes, hypertension and systemic inflammation. With regard to cholesterol, total cholesterol was assessed enzymatically in the serum (using the Cholesterol High Performance reagent; Roche Diagnostics, Indianapolis, IN), with elevated cholesterol considered above 200 mg/dL. With regard to weight status, participants were classified as overweight/obese based on a measured body mass index (BMI) of ≥25 kg/m^2^. Presence of diabetes was based on self-report of physician diagnosis of diabetes. Hypertension was defined as taking anti-hypertensive medications or a measured blood pressure of 140/90 mm Hg from the average of four blood pressure measurements. Lastly, systemic inflammation was defined as a high sensitivity C-reactive protein level >0.3 mg/dL, quantified using latex-enhanced nephelometry.

Behavioral

Behavioral parameters assessed included physical activity, dietary behavior, smoking status and sleep. Objectively-measured, free-living moderate-to-vigorous physical activity (MVPA) was assessed using an ActiGraph 7164 accelerometer, with participants wearing the monitor for up to 7 consecutive days. Only those with ≥4 days of ≥10 h/day of monitored data were evaluated. Activity counts/min ≥2020 were used to define MVPA.^12^ Nonwear time was identified as ≥60 consecutive minutes of zero activity counts, with allowance for 1-2 minutes of activity counts between 0 and 100.^13^

With regard to dietary behavior, two 24-hour recall assessments of food and fluid intake were collected during participant visits to a mobile examination center (MEC). To capture intake on all days of the week, the 24 hour recalls were collected on every day of the week. The dietary interviewers used the dietary data collection (DDC) system, which is an automated standardized interactive dietary interview and coding system. The Healthy Eating Index (HEI) 2005 was developed by the United States Department of Agriculture (USDA) as an indicator of dietary quality.^14^ The HEI is comprised of 12 components (total fruit; whole fruit; total vegetable; dark green, orange vegetable and legumes; total grain; whole grain; milk; meat and beans; oil; saturated fats; sodium; and calories from solid fats, alcoholic beverages, and added sugars) with each component individually scored, with a maximum total score of 100. A higher score reflects closer adherence to the dietary guidelines for Americans. The HEI was derived for each of the 24 hour recall days using the MyPyramid Equivalents database and following the methods and SAS code established by the USDA Center for Nutrition Policy and Promotion.^15-18^ Using the average of the two-day HEI scores, participants at or above the 60th percentile (i.e. top 40%) of HEI scores in the population were categorized as adhering to the dietary guidelines or consuming a healthy diet.^19^

Serum cotinine, a biological measure of smoking status,^20^ was measured by an isotope dilution-high performance liquid chromatography/atmospheric pressure chemical ionization tandem mass spectrometry. Serum cotinine levels of > 1.78 ng/mL for men and > 4.47 ng/mL for women were assessed to differentiate smokers from non-smokers.^20^

Participants were asked, “*How much sleep do you usually get at night on weekdays or workdays?,*” with hours slept per night categorized here as seven-to-nine or other. Notably, a modest (r=0.47) correlation has been observed between self-report and objectively-determined sleep duration.^21^

### Calculation of a PSBB index score

Fourteen PSBB parameters were evaluated herein, and thus, our calculated PSBB had a possible range of 0-14. As identified in the previous paragraphs, each of these PSSB parameters were coded as 0 or 1, where a score of “1” indicated a favorable score. For example, those who were depressed received a score of “0,” whereas those who met physical activity guidelines received a score of “1.”

In addition to creating an overall PSBB score, separate index scores were created for each construct within the PSBB index in order to evaluate their potential independent effects. That is, the *Psychological Index* included the participants depression score (0/1). The *Socioeconomic Index* included a score ranging from 0-4, including the variables of poverty level, education, race-ethnicity and living status. The *Biological Index* included a score ranging from 0-5, including the variables cholesterol, weight status, diabetes, hypertension and systemic inflammation. Lastly, the *Behavioral Index* included a score ranging from 0-4, including the variables smoking status, physical activity, sleep and diet.

### Analysis

Statistical analyses were performed via procedures from survey data using Stata (version 12); analyzed in 2015. To account for oversampling, non-response, non-coverage, and to provide nationally representative estimates, all analyses included the use of survey sample weights, clustering and primary sampling units. Cox proportional hazard models were used to examine the association between PSBB index and all-cause mortality. Covariates included age (years; continuous) and gender. Schoenfeld’s residuals were used to verify the proportional hazards assumption. Statistical significance was established as *P*<0.05.

## Results

### Participant characteristics

In the 2005-2006 NHANES cycle, 4979 adult (20+ years) participants were eligible for analysis. Among these, 2530 participants provided data on the PSBB parameters, with zero participants having missing mortality status data. These 2530 participants constituted the analytic sample.

Among the 2530 participants, 161 died over the follow-up period. The unweighted median follow-up period was 70.0 months (IQR=64-77); longest follow-up period was 83 months (6.9 years). In the sample, 175304 person-months occurred with a mortality incidence rate of 0.92 deaths per 1000 person-months. The weighted mean age of the sample was 46.3 years (SE=0.8; range=20-85 years), with 50.8% being female (Table 1).

**Table 1:** Characteristics of the analyzed sample, 2005-2006 NHANES (N=2530).

**Variable**	**Point Estimate**	**95% CI**
Age, mean years	46.2	44.5-47.9
Gender, % female	50.8	
Behavioral Index, mean	2.2	2.0-2.3
Socioeconomic Index, mean	3.2	3.1-3.3
Biological Index, mean	3.2	3.0-3.3
Depression, %	4.1	
PSBB Index, mean	9.5	9.3-9.7

Abbreviations: NHANES, National Health and Nutrition Examination Survey; PSBB, psycho-socioeconomic bio-behavioral.

### Characteristics of PSBB parameters

The weighted mean PSBB index score was 9.5 (SE=0.1; range=2-14). With regard to the individual index scores, among the 2530 participants, 132 were depressed (unweighted %, 5.2). With regard to the Socioeconomic Index, the range was 0-4, and the weighted mean was 3.19 (SE=0.1). For the Biological Index, the range was 0-5, and the weighted mean was 3.16 (SE=0.1). For the Behavioral Index, the range was 0-4, and the weighted mean was 2.17 (SE=0.1).

### Overall mortality results for PSBB

In an unadjusted Cox proportional hazard model, for every 1 increase in the overall PSBB, participants had a 21% reduced risk of all-cause mortality (HR=0.79; 95% CI: 0.71-0.87; *P*<0.001). After adjusting for age and gender, for every 1 increase in the overall PSBB, participants had a 15% reduced risk of all-cause mortality (HR = 0.85; 95% CI: 0.76-0.96; *P*=0.01); proportional hazards assumption was not violated (chi-square=0.19, df=2, *P*=0.97) and the Harrell’s C concordance statistic was 0.85. Adjusted results were similar when excluding those who died (n=12) within the first 12 months during the follow-up (HR=0.86; 95% CI: 0.77-0.95; *P*=0.007). Similarly, when excluding those with a physician diagnosis of coronary artery disease, stroke, congestive heart failure, heart attack or chronic obstructive pulmonary disease from the analysis (n=382), results were unchanged (HR=0.85; 95% CI: 0.77-0.93; *P*=0.002).

### Mortality results for PSBB components

In addition to examining the association of overall PSBB on all-cause mortality, we examined the association of each PSBB index on all-cause mortality. After controlling for age and gender, the Behavioral Index (HR=0.73; 95% CI: 0.60-0.88; *P*=0.003) and the Socioeconomic Index (HR=0.82; 95% CI: 0.68-0.99; *P*=0.04) were significant, but the Psychological Index (HR=0.67; 95% CI: 0.29-1.51; *P*=0.31) and the Biological Index (HR=1.03; 95% CI: 0.89-1.18; *P*=0.64) were not.

## Discussion

The intent of this manuscript was not to detail the effects of each component of the PSBB on mortality, as this has been extensively documented in the literature.^6-8,22^ The main findings of this brief report was the suggestive evidence of a cumulative PSBB risk on all-cause mortality. That is, those with a greater and more favorable PSBB index had a lower all-cause mortality risk. Further, the Behavioral Index and Socioeconomic Index were independent predictors of all-cause mortality. In a simplistic manner, this suggests that in adult populations, and in particular, socioeconomic vulnerable populations (e.g., low income, minorities), promotion of concurrent health behaviors (e.g., regular physical activity, healthy eating, smoking avoidance, and adequate sleep) may help to promote overall well-being and prolong survival. Although the Psychological and Biological indexes were not independently associated with all-cause mortality, the importance of these parameters should not be diminished given our observed association between the overall PSBB index and mortality, along with previous work demonstrating independent effects of these parameters on mortality.^1,3^ Encouragingly, however, concurrent adoption of health behaviors may not only influence survival, but may improve immediate health outcomes, including psychological well-being and favorable cardiometabolic functioning.^23-27^ Thus, based on the present study’s findings as well as those of others, practitioners are encouraged to promote a holistic health approach, particularly to vulnerable populations (e.g., those living in poverty) focusing on promoting health-enhancing behaviors that can have beneficial effects on psychological and biological parameters.

In conclusion, in this brief report, we set out to evaluate the potential cumulative association of PSBB risk on all-cause mortality. We indeed observed a cumulative PSBB risk on all-cause mortality. These findings align with other studies evaluating each individual PSBB component, and collectively, suggest the importance of maintaining overall health, including psychological, social, biological, and behavioral health. Despite the notable strengths of this study (e.g., national sample, which increases generalizability; novel study; several objective measures), future research is encouraged to overcome limitations of this study, which include the relatively short follow-up period as well as the inability to examine how trajectories of PSBB over time influence mortality. Additionally, the limited number of deaths precluded the ability to examine cause-specific mortality effects. Lastly, future work on this topic evaluating multiple psychological constructs is warranted.

## Funding

No funding was used to prepare this manuscript.

## Ethical approval

Procedures were approved by the National Center for Health Statistics review board; written consent was obtained prior to data collection.

## Competing interests

The authors declare no conflicts of interest.

## Authors contributions

PDL was involved in the conception of the study, performed the analyses and drafted the manuscript. RED was involved in the conception of the study, interpreted the results from the analyses, and assisted in the revision of the manuscript.

## References

[1] Cuijpers P, Smit F (2002). Excess mortality in depression: a meta-analysis of community studies. J Affect Disord.

[2] Caleyachetty R, Echouffo-Tcheugui JB, Shimbo D, Zhu W, Muennig P (2014). Cumulative social risk and risk of death from cardiovascular diseases and all-causes. Int J Cardiol.

[3] Di Angelantonio E, Kaptoge S, Wormser D, Willeit P, Butterworth AS, Bansal N (2015). Association of cardiometabolic multimorbidity with mortality. JAMA.

[4] Loprinzi PD (2016). Accelerometer-determined physical activity and mortality in a national prospective cohort study of adults at high risk of a first atherosclerotic cardiovascular disease event. Int J Cardiol.

[5] Dankel SJ, Loenneke JP, Loprinzi PD (2016). Physical activity and diet on quality of life and mortality: The importance of meeting one specific or both behaviors. Int J Cardiol.

[6] Loprinzi PD (2015). Dose-response association of moderate-to-vigorous physical activity with cardiovascular biomarkers and all-cause mortality: considerations by individual sports, exercise and recreational physical activities. Prev Med.

[7] Loprinzi PD (2015). Combined effects of accelerometer-assessed physical activity and dietary behavior on all-cause mortality in a national prospective cohort study. Int J Cardiol.

[8] Loprinzi PD (2015). Accumulated short bouts of physical activity are associated with reduced premature all-cause mortality: implications for physician promotion of physical activity and revision of current US government physical activity guidelines. Mayo Clin Proc.

[9] National Health and Nutrition Examination Survey. Centers for Disease Control and Prevention website. Available at: http://www.cdc.gov/nchs/nhanes.htm.

[10] Kroenke K, Spitzer RL, Williams JB (2001). The PHQ-9: validity of a brief depression severity measure. J Gen Intern Med.

[11] Caleyachetty R, Echouffo-Tcheugui JB, Muennig P, Zhu W, Muntner P, Shimbo D (2015). Association between cumulative social risk and ideal cardiovascular health in US adults: NHANES 1999-2006. Int J Cardiol.

[12] Loprinzi PD (2015). Objectively-measured physical activity and predicted 10-yr risk for a first atherosclerotic cardiovascular disease (ASCVD) event using the pooled cohort risk equations among US adults. Int J Cardiol.

[13] Troiano RP, Berrigan D, Dodd KW, Masse LC, Tilert T, McDowell M (2008). Physical activity in the United States measured by accelerometer. Med Sci Sports Exerc.

[14] Guenther PM, Reedy J, Krebs-Smith SM, Reeve BB, Basiotis PP. Development and evaluation of the Healthy Eating Index-2005: Technical report. Center for Nutrition Policy and Promotion, US Department of Agriculture; 2007. http://www.cnpp.usda.gov/HealthyEatingIndex.htm.

[15] United States Department of Agriculture. Center for Nutrition Policy and Promotion website. Available: http://www.cnpp.usda.gov/HealthyEatingIndex-2005report.htm.

[16] Bowman, S.A., Friday, J.E., Moshfegh, A. (2008). MyPyramid Equivalents Database, 2.0 for USDA Survey Foods, 2003-2004 [Online] Food Surveys Research Group. Beltsville Human Nutrition Research Center, Agricultural Research Service, U.S. Department of Agriculture, Beltsville, MD. Available at: http://www.ars.usda.gov/ba/bhnrc/fsrg.

[17] United States Department of Agriculture. Center for Nutrition Policy and Promotion. Other Projects. Available at: http://www.cnpp.usda.gov/OtherProjects.htm.

[18] National Cancer Institute. Healthy Eating Index - 2005 Tools for Researchers. Sample SAS code to create HEI-2005 scores. Available from: http://riskfactor.cancer.gov/tools/hei/tools.html.

[19] Ford ES, Bergmann MM, Boeing H, Li C, Capewell S (2012). Healthy lifestyle behaviors and all-cause mortality among adults in the United States. Prev Med.

[20] Benowitz NL, Bernert JT, Caraballo RS, Holiday DB, Wang J (2009). Optimal serum cotinine levels for distinguishing cigarette smokers and nonsmokers within different racial/ethnic groups in the United States between 1999 and 2004. Am J Epidemiol.

[21] Lauderdale DS, Knutson KL, Yan LL, Liu K, Rathouz PJ (2008). Self-reported and measured sleep duration: how similar
are they?. Epidemiology.

[22] Dankel SJ, Loenneke JP, Loprinzi PD (2016). Physical activity and diet on quality of life and mortality: The importance of meeting one specific or both behaviors. Int J Cardiol.

[23] Loprinzi PD, Mahoney S (2014). Concurrent occurrence of multiple positive lifestyle behaviors and depression among adults in the United States. J Affect Disord.

[24] Loprinzi PD (2013). Objectively measured light and moderate-to-vigorous physical activity is associated with lower depression levels among older US adults. Aging Ment Health.

[25] Loprinzi PD, Fitzgerald EM, Cardinal BJ (2012). Physical activity and depression symptoms among pregnant women from the National Health and Nutrition Examination Survey 2005-2006. J Obstet Gynecol Neonatal Nurs.

[26] Mahoney SE, Loprinzi PD (2014). Influence of flavonoid-rich fruit and vegetable intake on diabetic retinopathy and diabetes-related biomarkers. J Diabetes Complications.

[27] Loprinzi PD, Mahoney SE (2015). Association between flavonoid-rich fruit and vegetable consumption and total serum bilirubin. Angiology.

